# Supplementary material for: Pregnancy Associated Plasma Protein-A as a Cardiovascular Risk Marker in Patients with Stable Coronary Heart Disease During 10 Years Follow-Up—A CLARICOR Trial Sub-Study
Source: J Clin Med. 2020 Jan 18;9(1):265. doi: 10.3390/jcm9010265 (PMC7019890; doi:10.3390/jcm9010265)
Supplement: Supplementary file 1 [file jcm-09-00265-s001.pdf]

Journal of
Clinical Medicine

MDPI

Supplement to

Pregnancy associated plasma protein-A as a cardiovascular risk marker in patients with stable coronary heart disease during 10 years follow-up – a CLARICOR trial sub-study

Supplementary tables referenced in the main text are presented below.

Table S1. Logistic regression analyses of risk associated with elevated PAPP-A ≥ 4 mIU/L

Outcome	Variable	Discovery cohort	Replication cohort
**Composite outcome**	**Odds ratio**	1.46	1.13
	**95% CI**	1.06-1.99	0.82-1.56
	**p-value**	0.019	0.45
**All-cause mortality**	**Odds ratio**	1.66	1.64
	**95% CI**	1.22-2.26	1.18-2.27
	**p-value**	0.001	0.003

Shown are results from logistic regression models for the outcomes that violated the proportional hazard assumption for age at entry. Adjustment was for established risk factors and co-morbidities, standard biochemical predictors and treatments as shown in Supplementary Table 1, including age at entry. The composite outcome was defined as AMI, unstable angina pectoris, cerebrovascular disease, or death due to any cause. Abbreviations: PAPP-A, pregnancy-associated plasma protein A.

Table S2. Risk of specific outcomes associated with elevated PAPP-A

Outcome	Variable	Discovery cohort		Replication cohort	
		**Model A**	**Model B**	**Model A**	**Model B**
**Acute myocardial infarction**	**Hazard ratio**<br>**95% CI**<br>**p-value**	1.19<br>0.92-1.56<br>0.19	1.15<br>0.88-1.50<br>0.32	0.93<br>0.68-1.26<br>0.65	0.87<br>0.64-1.18<br>0.37
**Unstable angina pectoris**	**Hazard ratio**<br>**95% CI**<br>**p-value**	1.13<br>0.83-1.54<br>0.43	1.13<br>0.83-1.54<br>0.44	0.85<br>0.60-1.21<br>036	0.81<br>0.57-1.17<br>0.27
**Cerebrovascular disease**	**Hazard ratio**<br>**95% CI**<br>**p-value**	1.22<br>0.89-1.70<br>0.22	1.19<br>0.87-1.66<br>0.27	0.99<br>0.71-1.38<br>0.96	0.90<br>0.64-1.25<br>0.52
**Cardiovascular mortality**	**Hazard ratio**<br>**95% CI**<br>**p-value**	1.28<br>0.97-1.70<br>0.079	1.24<br>0.93-1.65<br>0.14	1.48<br>1.14-1.92<br>0.003	1.27<br>0.97-1.65<br>0.084
**Non-cardiovascular mortality**	**Hazard ratio**<br>**95% CI**<br>**p-value**	1.62<br>1.27-2.08<br><0.001	1.57<br>1.22-2.03<br><0.001	1.29<br>0.99-1.70<br>0.062	1.23<br>0.93-1.61<br>0.14
**All-cause* mortality**	**Hazard ratio**<br>**95% CI**<br>**p-value**	1.85<br>1.54-2.22<br><0.001	1.49<br>1.23-1.80<br><0.001	1.77<br>1.47-2.12<br><0.001	1.37<br>1.14-1.66<br>0.001

Results from Cox proportional hazards models are shown. Elevated PAPP-A was defined as baseline values ≥ 4 mIU/L and using those with PAPP-A <4 mIU/L as referents. Model A was adjusted for age at entry and sex. Model B was adjusted for established risk factors and co-morbidities, standard biochemical predictors and treatments as shown in Supplementary Table 1. * Proportional hazards assumption was violated for age at entry. Abbreviations: PAPP-A, pregnancy-associated plasma protein A.

Table S3. The role of elevated PAPP-A when used in combination with 'standard predictors' in the prediction of outcome status

Type of predictions	Standard predictors	PAPP-A added to 'standard predictors'
		**Composite outcome**
**True favorable predictions, N (%)**	2910 (48.7)	2906 (48.7)
**True unfavorable predictions, N (%)**	1174 (19.7)	1164 (19.5)
**Total number of true predictions, N (%)**	4084 (68.4)	4070 (68.2)
		**All-cause mortality**
**True favorable predictions, N (%)**	4585 (76.8)	4584 (76.8)
**True unfavorable predictions, N (%)**	392 (6.57)	405 (6.78)
**Total number of true predictions, N (%)**	4977 (83.4)	4989 (83.6)

Correct predictions of status of status of composite outcome (AMI, UAP, CVD, death) not experienced by patient (favorable status) versus experienced by patient (unfavorable status) and for the outcomes death (favorable status alive versus unfavorable status not alive) and correct predictions, in 2199 placebo patients. Predictions of status was made at 3 years, at 6 years, and at 9 years following randomization. In all, for the composite outcome 5972 predictions were made and for all-cause mortality 5971 predictions were made. The first model includes the standard predictors listed in **appendix A** as covariates and the second model includes the standard predictors plus binary PAPP-A as covariables.

© 2019 by the authors. Submitted for possible open access publication under the terms and conditions of the Creative Commons Attribution (CC BY) license (http://creativecommons.org/licenses/by/4.0/).
